# Phenolic profile, anti-inflammatory, antinociceptive, anti-ulcerogenic and hepatoprotective activities of *Pimenta racemosa* leaves

**DOI:** 10.1186/s12906-018-2260-3

**Published:** 2018-07-05

**Authors:** Fatma Abd-elkader Moharram, Amal Amin Al-Gendy, Siham Mostafa El-Shenawy, Bassant M. Ibrahim, Mohamed A. Zarka

**Affiliations:** 1 Department of Pharmacognosy, Faculty of Pharmacy, Helwan University, Cairo, 11795 Egypt; 2 Department of Pharmacognosy, Faculty of Pharmacy, Zagazig University, Zagazig, 44519 Egypt; 3 Department of Pharmacology, National Research Center, Dokki, Cairo, 12622 Egypt; 4 Department of Pharmacognosy, Faculty of Pharmacy, Modern Sciences and Arts University, 6 October City, Egypt; 5 Present Address: Department of Pharmacognosy, Faculty of Pharmacy, Modern University for Technology and Information, Cairo, Egypt

**Keywords:** Anti-inflammatory, Antinociceptive, Anti-ulcerogenic, Hepatoprotective, *Myrtaceae*, Phenolic compounds, *Pimenta racemosa*

## Abstract

**Background:**

*Pimenta racemosa* tree has many traditional uses where its leaves are used as herbal tea for treatment of flatulence, gastric disorder, osteoarthritis, colds and fever in addition to its analgesic and anti-inflammatory activities. So, this study aimed to isolate phenolic constituents of 80% aqueous methanol extract (AME) of leaves and evaluate its biological activities.

**Methods:**

The defatted AME was chromatographed and structures of the isolated compounds were elucidated using UV, NMR spectroscopy and UPLC-ESI-MS analysis. Antioxidant activity was investigated using 2, 2-diphenyl-1-picrylhydrazyl (DPPH) radical scavenging capacity. Anti-inflammatory activity was evaluated using carrageenan - induced paw oedema, while antinociceptive activity was determined by chemical and thermal stimuli. Anti-ulcerogenic effect of AME against gastric damage induced by ethanol in Wister male albino rats was evaluated. Also, hepatoprotective activity was investigated through determination of alanine aminotransferase (ALT) and aspartate aminotransferase (AST) following oral administration of paracetamol. Both of Anti-ulcerogenic and hepatoprotective activities (125, 250 and 500 mg/kg b.wt.) were supported by histopathological examinations.

**Results:**

Gallic acid (**1**), methyl gallate (**2**), avicularin (**3**), quercetin 3-*O*-β-*D*-arbinopyranoside (**4**), quercetin 3-*O*-*β*-D-glucopyranoside (**5**), quercetrin (**6**), cynaroside (**7**), strictinin (**8)**, castalagin (**9**), grandinin (**10**) quercetin (**11**) and ellagic acid (**12**) were isolated. AME showed significant radical scavenging activity (SC_50_ = 4.6 μg/mL), promising anti-inflammatory effect through inhibition of oedema and antinociceptive activity by reduction in number of writhes after acetic acid injection and prolongation of reaction time towards the thermal stimulus. AME reduced the gastric mucosal lesions compared with ethanol control and ranitidine groups, ALT at the three doses and AST only at 125 and 250 mg/kg b.wt., when compared with paracetamol group. The results were confirmed by histopathological studies.

**Conclusion:**

*P. racemosa* leaves are rich in phenolic compounds and showed significant biological activities.

## Background

*Pimenta racemosa* (Myrtaceae) is commonly known as the West Indian bay tree, bay rum tree and cilimnet*.* It is native to Carbbean region but it is also cultivated in many warm parts of the world. It is a large tree up to 25 ft. high [1]. The traditional uses and pharmacological properties of *P. racemosa* are mostly due to essential oil phenolic constituents, which have shown anti-inflammatory, analgesic, antimicrobial and antioxidant properties [2, 3]. The leaves were indicated for treatment of arthrosis, oedema, gastrosis, inflammation, myalgia and rheumatism in addition to other useful uses [4]. Tea prepared from the leaves of *P. racemosa* was used as a stimulant and for treatment of flatulence, colds and fever. Moreover, the leaf essential oil was used for treatment of both stomach pains, and externally for skin diseases [5] while the bark exhibited strong antischistosomal activity [6]. Antinociceptive and anti-inflammatory effects were also reported for the aqueous extract from leaves of *P. racemosa* var. *ozua* [7]. Few studies were conducted concerning the previously isolated compounds from *Pimenta* species. Galloyl glucosides and phenolic glycosides were isolated from *P. dioica* berries [8, 9], while galloyl glucosides, flavonoids, tannins and triterpenes were reported in *P. dioica* leaves [10, 11]. To date, there are no literature reports about the phenolic compounds and pharmacological activities of *P. racemosa* (Mill) J. W. Moore leaves. Inspired by *Pimenta dioica* leaves which are rich in flavonoids and phenolic compounds exhibited antioxidant and hepatoprotective activities in animal model [12], we were encouraged to investigate *P. racemosa* J.W. Moore leaves for the same effects. Therefore, our objective is to isolate the phenolic constituents from AME of leaves and evaluate its anti-inflammatory, antinociceptive, anti-ulcerogenic and hepatoprotective activities.

## Methods

### Plant material

Leaves of *P. racemosa* (Mill) J. W. Moore (syn. *Myrtus caryophyllata*, Lacq. Not L, *P. acris* Kostel), Myrtaceae were collected from Al- Zohria garden, Giza, Egypt during the flowering stage in April 2013. Permission of collection and authentication of the plant was performed by Dr. Trease Labib, former Head of El Orman Botanical Garden, Giza, Egypt which was found matching with the previously reported identification for this plant [6]. Voucher specimen (No.00099CP @ 04–07–04-01) has been deposited in the herbarium of Orman Botanical Garden, Giza, Egypt.

### Instruments and materials

The NMR spectral data were measured using JEOL GX-600 (500 and 100 MHz) and Bruker (400 and 100 MHz) for ^1^H and ^13^C-NMR, respectively. The results were reported as δ ppm values relative to TMS as internal reference. UV analyses were measured in MeOH and different UV shift reagents for pure samples using Shimadzu UV-VIS.1800 spectrophotometer. UV-visible spectrophotometer (Milton Roy, Spectronic 1201) was used for antioxidant activity. For column chromatography, polyamide S (Fluka Chemie AG, Switzerland), sephadex LH-20 (Sigma-Aldrich Steinheim, Germany) and microcrystalline cellulose (E. Merck-Darmstadt, Germany) were used. Whatman No.1 (Whatman Ltd., Maidstone, Kent, England) was used for paper chromatography. Ferric chloride spray reagent was used for visualization of tannins compounds, while Naturstoff reagent and aluminum chloride were used for flavonoids under UV light. *n*-BuOH/ HOAc/H_2_O; 4:1:5 *v*/v/v, top layer (S_1_) and 15% aqueous HOAc (S_2_) were used as solvent systems for determination of R_f_ values. Indomethacin, paracetamol and silymarin were obtained from Eipico, Misr and Sedico Companies, Egypt, respectively while ranitidine was obtained from Boehringer Ingelheim GmbH. Kits for alanine aminotransferase (ALT), aspartate aminotransferase (AST) and 2,2-diphenyl-1-picrylhydrazyl (DPPH) obtained from Sigma Chemical Company, USA were used for biological evaluation. All other chemicals and solvent are of high grade.

Compounds 9 and 10 molecular weights were estimated using UPLC-ESI-MS analysis (negative mode) via a UPLC Waters Corporation, Milford MA01757-U.S.A connected to XEVO TQD Triple Quadrupole Mass Spectrometry. Sample (10 μL) was injected into ACQUITY UPLC BEH C18 Column (130 Å, 1.7 μm, 2.1 mm × 50 mm). A binary gradient of 1% formic acid in deionized water (solvent A) and methanol containing 0.1% formic acid (solvent B) was constructed as follows: 50% solvent (A): 50% solvent (B). The flow rate was 1 mL/min. The mass spectrometer was fitted to an atmospheric pressure electrospray ionization (ESI) source, operated in negative ion mode. The electrospray capillary voltage was set to 3250 V, with a nebulizing gas flow rate of 9 L/h and a drying gas temperature of 450 °C. Mass spectrometry data were acquired in the scan mode (mass range *m*/*z* 100–1450). The instrument was controlled by Xcalibur software (Xcalibur™2.0.7, Thermo Scientific).

### Extraction and isolation

**Fig. 1:**
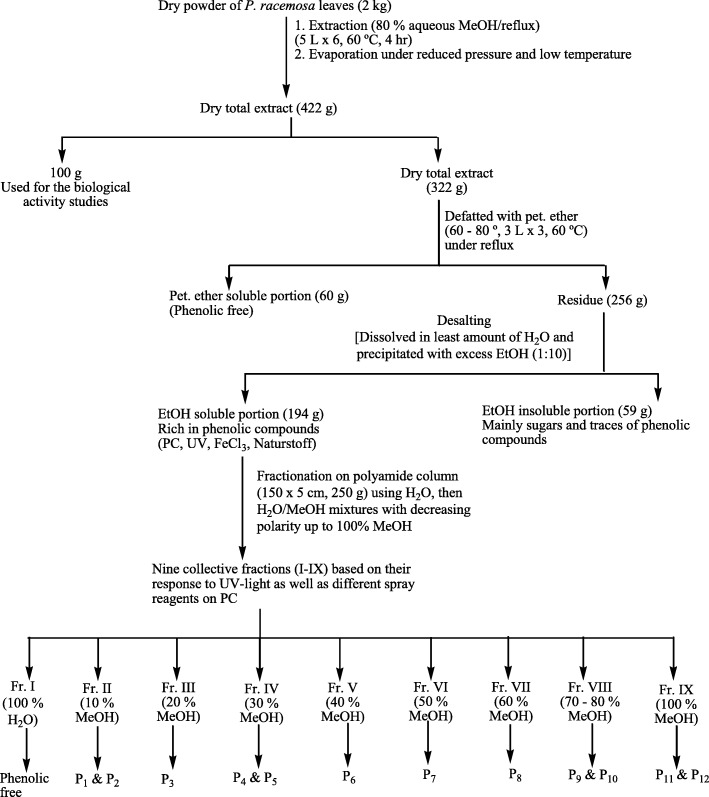
Extraction and fractionation of *P. racemosa* leaves total extract

Air dried powder of *P. racemosa* leaves was extracted and fractionated according to the procedures of Fig. 1 to afford nine collective fractions (I-IX).

Fraction II was fractionated on cellulose column using water/methanol (10–90%) mixture to give two sub-fractions, which were further purified on sephadex column using 50% methanol to give pure sample of 1 and 2. Each of fraction III, VI and VII was chromatographed on cellulose column using BIW (butanol: isopropyl alcohol: water, 4:1:5 top layer) followed by sephadex column using methanol/water (1: 1) to give pure samples of 3, 7 and 8 respectively. Fraction IV was purified on cellulose column using water/ methanol as an eluent to afford crude samples of 4 and 5 in two sub-fractions. They were further purified using sephadex column using methanol/water (1:1) as eluent to isolate chromatographically pure samples of 4 and 5. Fraction V was purified using successive columns of cellulose and sephadex (water/methanol mixtures) which led to a pure sample of 6. Fraction VIII was chromatographed on successive columns of cellulose using ethanol 20–70% and sephadex with BIW as an eluent to afford crude samples of 9 and 10, which were further purified through precipitation from their ethanolic solutions. Fraction IX was subjected to cellulose column using methanol/water mixture to afford crude samples of 11 and 12, which were purified by precipitation from their solutions.

### Biological activities

#### DPPH radical scavenging activity

Freshly prepared (0.004% *w*/*v*) methanol solution of 2,2-diphenyl-1-picrylhydrazyl (DPPH) radical was used to evaluate antioxidant activity. A 40 uL aliquot of the AME (2.5–640 μg/mL) was added to 3 mL of DPPH solution and the decrease in absorbance was measured at 515 nm [13]. The absorbance of the DPPH radical without antioxidant (control) and the reference compound ascorbic acid (5–80 μg/mL) were also measured. All the determinations were performed in three replicates and averaged. The percentage inhibition (PI) of the DPPH radical was calculated according to the formula:

$$ \mathrm{PI}=\left(\mathrm{AC}\hbox{-} \mathrm{AT}\right)/\mathrm{AC}\ \mathrm{x}\ 100 $$

Where AC = Absorbance of the control at *t* = 0 min and AT = absorbance of the sample + DPPH at *t* = 16 min.

The percentage of DPPH radical-scavenging was plotted against each applied concentration of AME and ascorbic acid to determine SC_50_ (the concentration required to scavenge DPPH by 50%).

### In vivo studies

#### Animals

Wister albino rats of both sexes (150-175 g) were used for acute toxicity while female ones were used for determination of the anti-inflammatory, anti-ulcerogenic and hepatoprotective activities and Swiss mice of both sexes (20-30 g) were used for determination of antinociceptive activity. The animals were obtained from the animal house colony of the National Research Centre, Dokki, Giza, Egypt. The animals were housed in standard metal cages in an air conditioned room at 22 ± 3°C, 55 ± 5% humidity and provided with standard laboratory diet and water *ad libitum.* Distilled water was used as a vehicle for all extracts and drugs used in the study. All animal procedures were carried out according to the Ethics Committee of the National Research Centre, Cairo, Egypt (Ethics NO. 17-021) and followed the guidelines of the National Institutes of Health Guide for Care and Use of Laboratory Animals.

For anti-ulcerogenic and hepatoprotective experiments, the animals were anesthetized using ether inhalation and euthanized by decapitation.

### Acute toxicity study

The AME was dissolved in distilled water and administered orally to 50 adult Wister albino rats of both sexes (150–175 g) in 5 graded doses up to 5 g/kg b.wt., while control group received the same volume of distilled water. Rats were observed for mortality. Experimental doses used were 1/40, 1/20 and 1/10 of 5 g / kg b.wt. of AME (125, 250 and 500 mg/kg b.wt for rats and 175, 350 and 700 mg/kg b.wt for mice).

### Anti-inflammatory activity

Paw oedema was induced by sub-plantar injection of right hind paw by 100 μl of 1% sterile lambda carrageenan suspension in saline [14]. The oedema component of inflammation was estimated by measuring hind foot pad immediately before carrageenan injection and 1–4 h post carrageenan injection with a planimeter [15]. Oedema was expressed as a percentage of change from control (pre-drug) values. Forty eight adult Female Wister albino rats (150–175 g) were divided into six groups (*n* = 8). 1st group received orally1 mL saline and used as control while 2nd group was sub-plantar injected in right hind paw with 100 μL of 1% carrageenan. 3rd group was given indomethacin (25 mg/kg b.wt.) orally as a reference anti-inflammatory drug. The remaining groups received orally 125, 250 and 500 mg/kg. b.wt. of the AME. All treatments were given orally 60 min before the injection of the carrageenan suspension.

### Antinociceptive effect

It was evaluated by measuring the responses of animals to both a chemical (acetic acid) and a thermal (hot plate) stimulus.

### Chemical test

Induction of writhing in mice using acetic acid was done according to Collier et al. [16]. 30 Swiss mice of both sexes (20–30 g) were divided into five groups each of six. The 1st control group received saline, while 2nd group received an oral dose of 200 mg/kg b.wt. of aspirin. 3rd- 5th groups received an oral dose of AME of *P. racemosa* leaves (125, 250 and 500 mg/kg b.wt.). After 30 min, the mice of all groups were injected *i.p* with 0.3% acetic acid (0.2 mL / mice) and placed in individual cages then the number of writhes was counted and compared in 30 min period.

### Thermal test

Thermal test was carried out using an electronically controlled hot-plate adjusted to 52 °C ± 0.1 °C and the cut-off time was 60s [17]. 30 Swiss mice of both sexes (20–30 g) were divided into five groups of mice each of six and they received the same doses as above mentioned in chemical test except the 2nd group was given 20 mg/ kg b.wt. of tramadol orally [18]. The time elapsed until either mice paw licking or jumping occurred was recorded 1 h before and after 1 and 2 h post treatment.

### Anti-gastroulcerogenic effect

Gastric lesions were induced in rats by oral administration of 1 mL 70% ethanol single dose [19]. Thirty six adult Female Wister albino rats (150–175 g) were divided into six groups each of six. 1st group received a daily oral dose of 1 mL distilled water (control group) while 2nd group given orally 1 mL of 70% ethanol. 3^rd^group was orally given 50 mg/kg b.wt. ranitidine as a reference drug [20] daily for two successive weeks. The remaining groups orally received AME (125, 250 and 500 mg/kg b.wt.) daily, also for two successive weeks. Ethanol was administered one hour after extract or ranitidine. At the end of the experiment, one hour after the last dose, the rats were sacrificed by cervical dislocation after being lightly anaesthetized. The stomach was excised, opened along the greater curvature, washed with saline and examined thoroughly for mucosal lesions. The number and severity of mucosal lesions were observed and lesions were scaled as follows; petechial lesions = 1, lesions < 1 mm = 2, lesion between 1 and 2 mm = 3, lesions between 2 and 4 mm = 4, lesions more than 4 mm = 5. A total lesion score for each animal was calculated by multiplication of the total number of lesions by the respective severity scores. Results are expressed as the severity of lesions per rat [21]. For histopathological study, the stomach was fixed in neutral buffered 10% formal saline for 72 h, rinsed in tap water and then dehydrated in alcohol (70–95%), cleared in xylene, impregnated in soft paraffin wax at 55 °C and finally embedded in hard paraffin. Serial sections of 6 μm in thickness were cut and stained with haematoxylin and eosin (H&E) [22].

### Hepatoprotective activity

Induction of hepatic damage was done by giving paracetamol orally [23]. Thirty six adult Female Wister albino rats (150–175 g) were divided into six groups each of six. 1st normal control group orally received 1 mL distilled water while 2nd group received a single oral dose of paracetamol (640 mg/kg b. wt.). 3rd-5th group were given a daily oral dose of AME (125, 250 and 500 mg/kg b. wt., respectively) alone for 15 successive days before paracetamol oral administration and the 6th group was given a daily oral dose of silymarin as a reference drug (25 mg/kg b.wt.) [24] also alone for 15 successive days before paracetamol oral administration. 24 h after paracetamol administration, the blood was obtained from rato-orbital plexus after anesthesia of rats [25] and centrifuged at 2500 rpm for 15 min. The serum was separated and collected for the determination of ALT and AST. For histopathological study, the liver specimens of all rats were dissected, fixed in 10% neutral-buffered normal saline for 72 h, washed with water and then dehydrated in grades of alcohol (70–95%), cleared in xylene and embedded in paraffin. Serial sections of 6 μm thick were cut and stained with haematoxylin and eosin [22].

### Statistical analysis

Values were expressed as means ± S.E. Comparisons between means were carried out using one way analysis of variance (ANOVA) followed by Tukey Kramer multiple comparisons test for all tests except for the thermal test which was conducted using hot plate and the anti-inflammatory test where comparisons between means were carried out using two way ANOVA followed by Bonferroni’s multiple comparisons test. *P* ≤ 0.05 was accepted as being significant in all types of statistical tests. Graph pad prism software (version 6) was used to carry out all statistical tests.

## Results

### Chromatographic and spectroscopic data of the isolated compounds

**Fig. 2:**
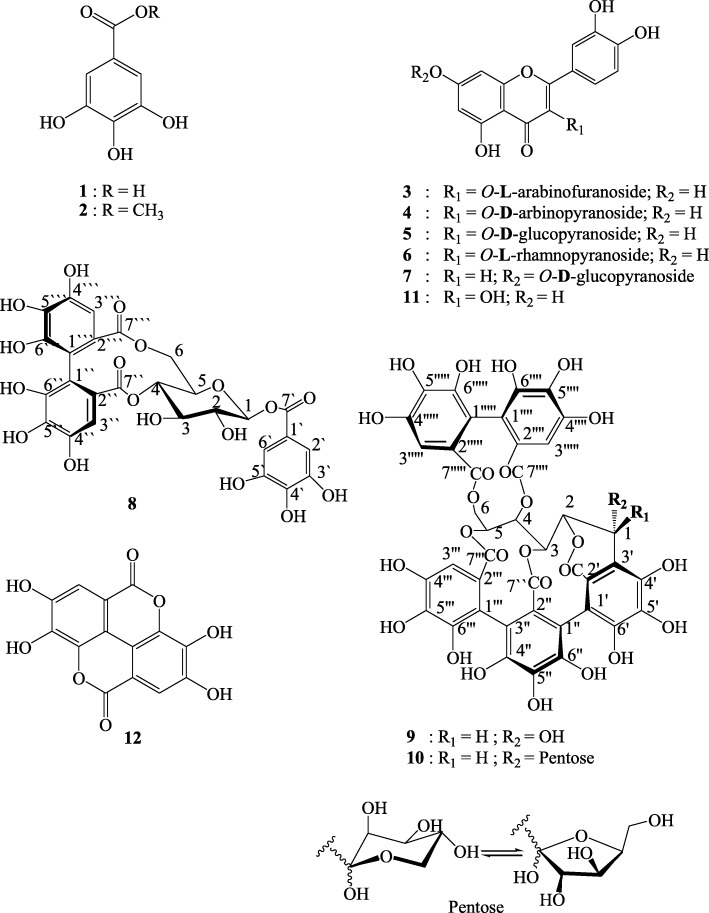
Structure of the compounds isolated from *P. racemosa* leaves AME

Two dimensional paper chromatography (2D-PC) screening of *P. racemosa* leaves’ AME revealed the presence of a mixture of phenolic compounds based on their color properties under UV-light and their response to different spray reagents. Fractionation of AME on a polyamide column, followed by purification on cellulose and Sephadex LH-20 columns resulted in the isolation of 12 compounds. Five flavonols, one flavone, three tannins in addition to two phenolic acids and one of their derivatives were isolated and identified*.* All compounds were isolated from *P. racemosa* leaves for the first time while compounds 3–7 were isolated from genus *Pimenta* for the first time. The structures of these compounds (Fig. 2) were fully elucidated on the basis of their physicochemical and spectral data (UV, ^1^H-NMR, ^13^C-NMR and UPLC-ESI-MS) and by comparison with published data and authentic samples.

Both of compounds **1** (45 mg) and **2** (27 mg) were obtained as off-white amorphous powder. They gave shine violet fluorescent spots under short UV- light and blue color with Fe Cl_3_ spray reagent. R_f_ values are 0.14 **(S**_**1**_**)** and 0.75 (**S**_**2**_) for **1** and 0.18 **(S**_**1**_**)** and 0.69 (**S**_**2**_) for **2**. The identification was based upon Co-PC with available authentics and comparison of spectroscopic data with the matched literature as will be discussed later.

Compounds **3** (22 mg), **4** (17 mg), **5** (20 mg) and **6** (25 mg) were purified as yellow amorphous powder showing deep purple fluorescence under long UV. They gave green and orange colour when sprayed by ferric chloride and Naturstoff reagents, respectively. Their R_f_ values, UV, ^1^H and ^13^C-NMR data are listed in Tables 1 and 2.

**Table 1:** R_f_ values and UV data of compounds 3–6

Compound	R_f_ values		UV data λ_max_ (nm)					
	S_1_	S_2_	MeOH	+ NaOMe	+ NaOAc	+NaOAc + H_3_BO_3_	+ AlCl_3_	+AlCl_3_ + HCl
3	0.5	0.52	256, 295 (sh), 352	269, 325 (sh), 403	270, 320 (sh), 367	269, 310, 402	270, 306 (sh), 335 (sh), 419	267, 293 (sh), 358 (sh), 395
4	0.47	0.54	254, 290 (sh), 354	270, 329 (sh), 410	271, 317 (sh), 372	270, 299, 405	273, 320 (sh), 410	272, 290, 365 (sh), 399
5	0.41	0.6	255, 264 (sh), 358	273, 409	270, 375	268, 293 (sh), 375	259 (sh), 265, 271, 409	252 (sh), 269, 365
6	0.43	0.55	252, 299 (sh), 355	265, 322 (sh), 415	269, 320 (sh), 368	264, 305 (sh), 377	272, 302 (sh), 315, 420	269, 305 (sh), 399

**Table 2:** ^1^H and ^13^C-NMR data of compounds 3–6

**C** No.	3		4		5		6	
	3 DMSO-*d6*, 500 and 125 MHz		4 (CD_3_OD-*d4*, 400 and 100 MHz)		5 DMSO-*d6*, 400 and 100 MHz		6 DMSO-*d6*, 400 and 100 MHz	
	δ _H_	δ_**C**_	δ _**H**_	δ_**C**_	δ _**H**_	δ_**C**_	δ _H_	δ_**C**_
2		157.49		157.39		157.00		157.61
3		133.85		134.21		133.58		134.57
4		178.19		178.10		177.48		178.07
5		161.72		161.59		161.56		161.73
6	6.16 *d* (1.9)	99.19	6.23 *br s*	98.57	6.11*br s*	99.83	6.17*br s*	99.36
7		164.83		164.62		167.27		165.50
8	6.37 *d* (1.9)	94.08	6.43 *br s*	93.39	6.31 *br s*	94.34	6.35 *br s*	94.18
9		156.86		157.00		156.24		156.97
10		104.42		104.23		103.44		104.23
1’		122.24		121.71		121.96		121.54
2’	7.44 *d* (1.95)	116.04	7.76 *d* (1.6)	114.85		115.72	7.29 *br s*	115.91
3’		145.59		144.58		145.46		145.71
4’		148.99		148.57		149.34		148.99
5’	6.82 *d* (8.3)	115.98	6.90 *d* (8.4)	116.09	6.83 *d* (8.4)	116.51	6.88 *d* (8.4)	115.72
6’	7.51 *dd* (1.95, 8.3)	121.43	7.59 *dd* (1.6, 8.4)	121.44		121.39	7.25 *dd* (*br s,* 8.4)	121.13
2’/6’					7.58 *m*			
H-1”	5.55*br s*	108.29	5.14 *d* (6.0)	103.22	5.44 *d* (6.8)	101.57	5.25 *br s*	102.25
H-2”	4.11 *m*	82.63	3.94–3.46 *m* remaining sugar protons	72.71	3.6–3.17 *m*, remaining sugar protons	74.58	3.53 *dd* (3.2, 2.0) 3.4-	70.79
H-3”	3.49–3.29 *m*, remaining sugar protons	77.39		71.46		76.98	3.1 *m*, remaining sugar protons	71.62
H-4”		86.28		67.75		70.36		71.04
H-5”		62.56		65.63		77.93		70.51
H-6”						61.40	1.09 *d* (6.1)	17.96

J-values (Hz) were reported in parenthesis

**Compound 7:** Yellow amorphous powder (20 mg) with R_f_ values 0.42 (**S**_**1**_) and 0.3 (**S**_**2**_) and giving dark purple spot under long UV that changed to orange fluorescence and pale green color with Naturstoff and FeCl_3_ spray reagents, respectively. UV spectral data λ_max_(nm) MeOH: 255, 269 (sh), 345; (+NaOMe): 258300 (sh), 400; (+NaOAc): 254 (sh), 268, 358 (sh), 390; (+NaOAc/H_3_BO_3_): 260, 294 (sh), 365 (+AlCl_3_): 265, 300 (sh), 430; (+AlCl_3_/HCl): 270, 283 (sh), 365.^1^H-NMR (400 MHz, DMSO, *d*_*6*_**)** δ ppm 13.00 (1H, *s*, H-bonded OH-5), 7.45 (2H, *m* H2’/6’), 6.90 (1H, *d*, J = 8.0, H-5’), 6.79 (1H, *s*, H-3), 6.77 (1H, *d*, J = 1.2, H-8), 6.45 (1H, *d*, J = 1.2, H-6), 5.09 (1H, *d*, J = 6.8 Hz, H-1”) and 3.72–3.24 (m, remaining sugar protons). ^13^C-NMR (100 MHz, DMSO, *d*_*6*_) 182.36 (C-4), 164.94 (C-2), 163.42 (C-7), 161.60 (C-5), 157.41 (C-9), 150.39 (C-4’), 146.25 (C-3’), 121.85 (C-1’), 119.63 (C-6’), 116.45 (C-2’), 114.04 (C-5’), 105.80 (C-10), 103.64 (C-3), 100.36 (C-6), 100.00 (C-1”), 95.19 (C-8), 77.63 (C-5”), 76.86 (C-3”), 73.58 (C-2”), 70.02 (C-4”) and 61.08 (C-6”).

**Compound 8:** Brown amorphous powder (15 mg) with R_f_ values 0.27 (**S**_**1**_) and 0.36 (**S**_**2**_); It gave dark purple fluorescent spot under short UV-light, turned to indigo-red and deep blue color with NaNO_2_-glacial AcOH and FeCl_3_ spray reagents, respectively. UV λ_max_(nm); MeOH: 246, 288 sh. ^1^H-NMR (400 MHz, DMSO-*d*_*6*_: δ ppm 6.97 (2H, s, H-2’/6’ galloyl moiety), 6.42 (1H, *s*, H-3”’HHDP), 6.40 (1H, *s*, H-3” HHDP), 5.70 (1H, *d*, J = 8.4 Hz, H-1), 4.86 (1H, *dd*, J = 12.8, 5.2. Hz, H-6_a_), 4.60 (1H, t-like, J = 10 Hz, H-4), 4.40 (1H, *br dd*, J = 12.8, 9.2 Hz, H-5), 3.68 (1H, *br d,* J = 14.0 Hz, H-6_b_), 3.63 (1H, t-like, J = 7.6 Hz, H-3) and 3.26 (1H, t-like, J = 10.0 Hz, H-2). ^13^C- NMR (100 MHz, DMSO-*d*_*6*_): δ ppm 167.83, 167.09 (C-7”’/7”HHDP), 164.62 (C-7’ G), 145.62 (C-3’/5’ G), 144.50, 144.41 (C-6”/6”’ HHDP), 144.26. 144.23 (C-4”/4”’ HHDP), 139.17 (C-4’ G), 135.33, 134.96 (C-5”/5”’ HHDP), 124.70, 124.42 (C-2”/2”’ HHDP), 118.42 (C-1’ G), 115.55, 115.23 (1”/1”’ HHDP), 109.09 (C-2’/6’ G), 106.29, 105.51 (C-3”/ 3”’ HHDP), 94.73 (C-1), 73.82 (C-3), 73.33 (C-5), 71.61 (C-4/2) and 62.74 (C-6).

**Compound 9:** It was obtained as brown amorphous powder (25 mg) with R_f_ value 0.59 (S_2_). It also showed dark purple fluorescent spot under short UV-light and dull brown under long UV-light, turned to indigo-red with NaNO_2_ - glacial AcOH and deep blue color with FeCl_3_, respectively. UV λ_max_(nm); MeOH: 248, 290 (sh). ^1^H-NMR (400 MHz, CD_3_OD-*d*_*4*_), δ ppm 6.69 (2H, *s*, H-3”’ flavogalonic acid (FLG); H-3”” HHDP), 6.50 (1 H, *s*, H-3””’ HHDP), 5.51 (1H, *d*, J = 4.0 Hz, H-1), 5.48 (1H, *d*, J = 8.0 Hz, H-5), 5.07 (1H, t-like, J = 8.0 Hz, H-4), 4.93 (1H, *dd*, J = 4.0, 12.0 Hz, H-6), 4.82 (2H, *m*, H-3/2 hidden by water signal) and 3.88 (1H, *d*, J = 12.0 Hz, H- 6’). ^13^C-NMR (100 MHz, CD_3_OD-*d*_*4*_): δ ppm 169.01, 166.87 (C-7””/C-7””’ HHDP), 166.15 (C-7”’ FLG), 165.01 (C-7” FLG), 164.96 (C-7’ FLG), 146.08 (C-4”’ FLG), 144.79, 144.68 (C-6””/6””’ HHDP), 144.66, 143.92 (C-4””/4””’ HHDP, 4’/4” FLG), 143.58, 142.27, 143.05 (C-6’/6”/6”’ FLG), 136.96 (C-5”’ FLG), 136.51, 136.40 (C-5””/5””’ HHDP), 135.87 (C-5” FLG), 134.65 (C-5”’ FLG), 126.63 (C-2” FLG), 125.43, 123.99 (C-2””/2””’ HHDP), 123.83 (C-2”’ FLG), 120.68 (C-2’FLG), 116.02, 115.28, 114.99, 114.45, 113.85, 113.78 (C-1””/1””’ HHDP, C-1’/1”/1’” FLG, C-3’ FLG), 112.33 (C-3” FLG), 108.22, 107.70, 106.93 (C-3”’ FLG, C-3””/3””’ HHDP), 73.68 (C-2), 70.77 (C-5), 68.63 (C-4), 66.35 (C-1), 65.55 (C-3) and 64.64 (C-6).

**UPLC-ESI-MS (negative mode),** *m/z* (relative abundance %): 933 ([M-H]^−^, 100%).

**Compound 10:** It was purified as brown amorphous powder (30 mg) with R_f_ value 0.61 (S_2_). It also gave dark purple fluorescent spot under short UV-light and dull brown under long UV-light, turned to indigo-red with NaNO_2_ - glacial AcOH and deep blue color with FeCl_3_, respectively. UV λ_max_(nm); MeOH: 245, 293 (sh). ^1^H-NMR (400 MHz, CD_3_OD, *d*_*4*_): δ ppm 7.32, 6.79, 6.79, 6.79, 6.78 (1H in total, each s, H-6”’), 6.63, 6.61, 6.62, 6.59 (2H in total, each s, H-6””/6””’), 5.78, 5.63, 5.61, 5.52, 5.23, 5.21, 5.20, 5.18, 5.16, 5.14 (2H in total, *m*, H-2/5), 4.87(H-4 hidden by H_2_O signal), 4.53 (1H, *dd*, J = 8.0, 16.0, Hz, H-6_a_), 4.28 (1H, *br d*, J = 8 Hz, H-3), 4.01, 3.98 (1H, in total, each *br s*, H-1), 3.90–3.32 (4H, m, H-6_b_, H-4 _pentoses_, 2H-5 _pentoses_). ^13^C-NMR (100 MHz, CD_3_OD- *d*_*4*_): δ ppm 169.23, 169.04 (C-7””’), 166.77, 166.53 (C-7”’), 166.37 (C-7””), 165.78 (C-7”), 165.78, 164.72, 164.74 (C-7’), 148.14, 146.73, 146.33, (C-5”’), 144.78, 144.74 (C-3””, 3””’), 144.69 (2C, C-5””, 5’””), 144.31, 143.89, 144.81, 143.57, 143.49 (C-5”, 5’), 143.39, 143.37, 143.31, 142.64 (C-3’, 3”, 3’”), 137.73, 137.28, 137.18 (C-4’”), 136.89, 136.61, 136.39, 136.29, 135.80 (C-4””, 4’””), 135.62, 134.70 (C-4”), 134.67 (C- 4’), 126.66, 126.50, 126.39 (C-1”), 125.63, 125.49, 125.44, 125.24 (C-1””, 1’””), 123.89, 123.84 (C-1”’), 123.42 (C-1′), 115.72, 115.40, 114.90, 114.58, 114.41, 114.18, 114.12, 113.87, 113.84, 113.03 (C-2’, 2”, 2’, 2””, 2’””), 112.26 (C-6’), 112.14, 110.40, 109.21 (C-6”), 108.04, 107.80, (C-6’”), 106.78, 106.53, (C-6””, 6’””). Sugar moieties: 101.15,101.07, 99.42, 99.34 (C-1 _pentose_), 80.08, 79.3, 72.09, 69.10 (C-4 _pentose_), 74.87, 74.38 (C-2 _pentose_), 73.46 (C-3 _pentose_), 71.87, 71.50 (C-2), 71.43, 71.38 (C-5), 70.64, 70.47, 70.29, 69.96 (C-4), 69.49, 69.00, 68.97, 68.38 (C-3), 66.25, 66.22, 64.67 (C-5_pentose_), 64.63, 62.30, 62.33 (C-6), 47.03, 46.96 and 45.86 (C-1).

**UPLC-ESI-MS (negative mode),** *m/z* (relative abundance %): 1065 ([M-H]^−^, 100%).

**Compound 11:** It was obtained as yellow amorphous powder (18 mg) with R_f_ value 0.75 (**S**_**1**_) and giving dark purple spot under long UV that changed to orange fluorescence and pale green color with Naturstoff and FeCl_3_ spray reagents, respectively.

**Compound 12:** It was obtained as off white amorphous powder (15 mg) with R_f_ value 0.25 **(S**_**1**_**)**. It gave shine violet fluorescent spots under short UV- light and blue color with Fe Cl_3_ spray reagent. The identification was based upon Co-PC with available authentics and comparison of spectroscopic data with the available literature as will be mentioned later.

### Biological activities

The DPPH scavenging percentages of AME and ascorbic acid as well as SC_50_ values indicated their significant antioxidant activities. AME showed promising antioxidant activities as indicated by its high DPPH scavenging percentage (89.67%) at 320 μg/mL and low SC_50_ value (4.6 μg/mL) when compared with the activity of the standard ascorbic acid (92.48% scavenging percentage at 40 μg/mL and SC_50_ = 14.2 μg/mL).

Acute toxicity study revealed that AME of *P. racemosa* is non-toxic up to 5 g/kg b.wt. After 24 h, there is no mortality recorded, and as Semler et al. reported [26], if just one dose level at 5 g/kg b.wt. is not lethal to animals, there is no need to determine LD_50_.

Concerning the anti-inflammatory activity, oral administration of 125 mg/kg b.wt. AME exhibited significant inhibition of oedema formation by 42.1, 45.01 and 26.68% after 2nd, 3rd and 4th h, respectively after carrageenan injection as compared with saline group at the same time post carrageenan injection, while 250 and 500 mg/ kg b.wt. induced significant inhibition of oedema formation by 23.34, 75.66, 76.87 and 50.66% for 250 mg/kg b.wt. and 61.46, 80.53, 86.12 and 93.83% for 500 mg/kg b.wt. after 1st, 2nd, 3rd and 4^th^hr, respectively post carrageenan injection in comparison to saline group. Moreover, administration of 500 mg/kg b.wt. of AME exhibited significant inhibition of oedema which exceeded the activity of indomethacin after 1st, 2nd, 3rd and 4th h that was indicated by the percentage of inhibition values (Table 3).

**Table 3:** Anti-inflammatory activity of *P. racemosa* leaves AME and indomethacin on carrageenan-induced paw oedema in rats

Oedema									
Drug (mg/kg b.wt. P.O)	Zero time (cm)	1 h (cm) (% increase)	% of Inhibition	2 h (cm) (% increase)	% of Inhibition	3 h (cm) (% increase)	% of Inhibition	4 h (cm) (% increase)	% of Inhibition
Saline	0.37 ± 0.01	0.49 ± 0.01	–	0.56 ± 0.03	–	0.57 ± 0.009	–	0.52 ± 0.02	–
		(32.43 ± 0.3)		(51.35 ± 0.54)^*^		(54.05 ± 0.63)^*^		(40.54 ± 0.6)	
Indomethain	0.36 ±0.01	0.48 ± 0.02	2.68	0.45 ± 0.02	51.31	0.44 ± 0.006	58.88	0.4 ± 0.01	72.59
(25)		(33.3 ± 0.21)^*^		(25 ± 0.25)^*^		(22.22 ± 0.22)^*^		(11.11 ± 0.38)^*^	
AME									
(125)	0.37 ± 0.01	0.47 ± 0.007	16.65	0.48 ± 0.02	42.10	0.48 ± 0.02	45.01	0.48 ± 0.02	26.68
		(27.03 ± 0.24)^*#^		(29.73 ± 0. 5)^*#^		(29.73 ± 0.54)^*#^		(29.73 ± 0.49)^*@#^	
(250)	0.4 ± 0.006	0.56 ± 0.03	23.34	0.45 ± 0.02	75.66	0.45 ± 0.02	76.87	0.48 ± 0.04	50.66
		(40 ± 1.9) ^#^		(12.5 ± 0.62)^*^		(12.5 ± 0.23)^*^		(20 ± 0.29)* ^#^	
(500)	0.4 ± 0.002	0.45 ± 0.01	61.46	0.44 ± 0.01	80.53	0.43 ± 0.02	86.12	0.41 ± 0.02	93.83
		(12.5 ± 3.4)^*@^		(10 ± 0.32)^*@^		(7.5 ± 0.33)^*@^		(2.5 ± 0.16)^*^	

Results are expressed as means % of inhibition ± SE; *n* = 8; *P* ≤ 0.05; % of increase of inflammation was measured relative to zero time of each group; % of inhibition of inflammation measured at the same time interval for each group)

^*^Significant difference from saline control group at *P* ≤ 0.05; ^@^ Significant different from indomethacin group at *P* ≤ 0.05; ^#^ Significant different from AME 500 mg/kg b.wt. group at *P* ≤ 0 .05

Regarding the antinociceptive activity either by chemical (acetic acid test) or thermal (hot-plate test) stimuli, it was found that AME caused significant decrease in the number of writhes in mice after acetic acid injection in dose-dependent manner being 18.66, 40.00 and 49.33% at 175, 350 and 700 mg/kg b.wt., respectively upon comparison with saline control (Table 4). AME (700 mg/kg b.wt.) showed a significant prolongation of the reaction time to the thermal stimulus by 23.28 and 45.15% after 1 and 2 h, respectively as compared with control pre-drug (Table 5).

**Table 4:** Antinociceptive activity of *P. racemosa* leaves AME and aspirin on acetic acid induced writhing in mice

Groups	Dose mg/kg b.wt.	Number of writhing / 30 min (X ± S. E)	% Inhibition of writhing	Potency
Saline	1 ml	15 ± 1.14	–	
Aspirin	200	5 ± 0.44^*^	66.66	1
AME	175	12.2 ± 0.58^*@^	18.66	0.28
	350	9 ± 0.31^*@#^	40	0.6
	700	7.6 ± 0.4^*@#^	49.33	0.74

Data represent the mean value ± S.E; *n* = 5

^*^Significant different from saline control group at *P* ≤ 0.05; ^@^ Significant different from aspirin group a *P* ≤ 0.05,^#^ Significant difference from the extract 175 mg/kg b.wt. group a *P* ≤ 0.05; Potency was calculated as regard the percentage change of the aspirin

**Table 5:** Antinociceptive activity of *P. racemosa* leaves AME and tramadol on thermal pain in mice

Group Dose (mg/kg b.wt.)	basal	1 h			2 h		
	Latency (s)	Latency (s)	Change %	Potency	Latency(s)	Change %	Potency
Saline	23.84 ± 0.27	23.07 ± 0.6	3.23	–	19.48 ± 0.62	18.29	–
Tramadol (20)	23.21 ± 0.81	22.72 ± 0.9	2.11	1	31.48 ± 0.4*^@^	35.63	1
AME							
175	23.01 ± 0.7	22.67 ± 0.81	1.48	0.7	24.46 ± 0.2	6.3	0.17
350	23.60 ± 0.42	23.52 ± 0.73	0.34	0.2	25.82 ± 0.7	9.4	0.26
700	22.90 ± 0.5	28.23 ± 0.8*^@^	23.28	11	33.24 ± 0.44*^@^	45.15	1.2

Data represent the mean value ±. S.E. (n + 6); *n* = 6

^*^Significantly difference from normal group at same time interval measurement P ≤ 0.05; ^@^ Significantly different from corresponding group at zero time interval measurement *P* ≤ 0.05; Potency was calculated as regard the percentage change of the tramadol

Concerning gastric ulcerogenic effect both the number and severity of gastric mucosal lesions induced by ethanol were reduced after oral administration of AME (125, 250 and 500 mg/kg) by 46.9& 52.8%, 79 & 82.4 and 82.7% & 91.5%, respectively relative to the control values while ranitidine showed reduction in the number and severity by 87.7 & 94.4%, respectively by comparison with ethanol groups (Table 6).

**Table 6:** Effect of *P. racemosa* leaves AME and ranitidine on gastric mucosal injury induced by ethanol in rats

Treated groups	Dose mg/kg b.wt.	Number of lesions/rat	% change	Severity of lesions/rat	% change
Ethanol (70%)	1 mL	16.20 ± 0.37	–	56.8 ± 0.97	–
Ranitidine	50	2.0 ± 0.31^*^	87.7	3.2 ± 0.37*	94.4
AME	125	8.6 ± 0.24^*@^	46.9	26.8 ± 0.37*^@^	52.8
	250	3.4 ± 0.24*^@#^	79	10 ± 0.44*^@#^	82.4
	500	2.8 ± 0.37^*#^	82.7	4.8 ± 0.37*^#^	91.5

Data is represented as means ± S.E; *n* = 5; Data were analyzed using one way analysis of variance (ANOVA) followed by Tukey Kramer multiple comparisons test; Significant at *P* ≤ 0.05

^*^Significant difference from positive control ethanol group; ^@^ Significant different from ranitidine group; ^#^ Significant difference from the extract (125 mg/kg) group

**Fig. 3:**
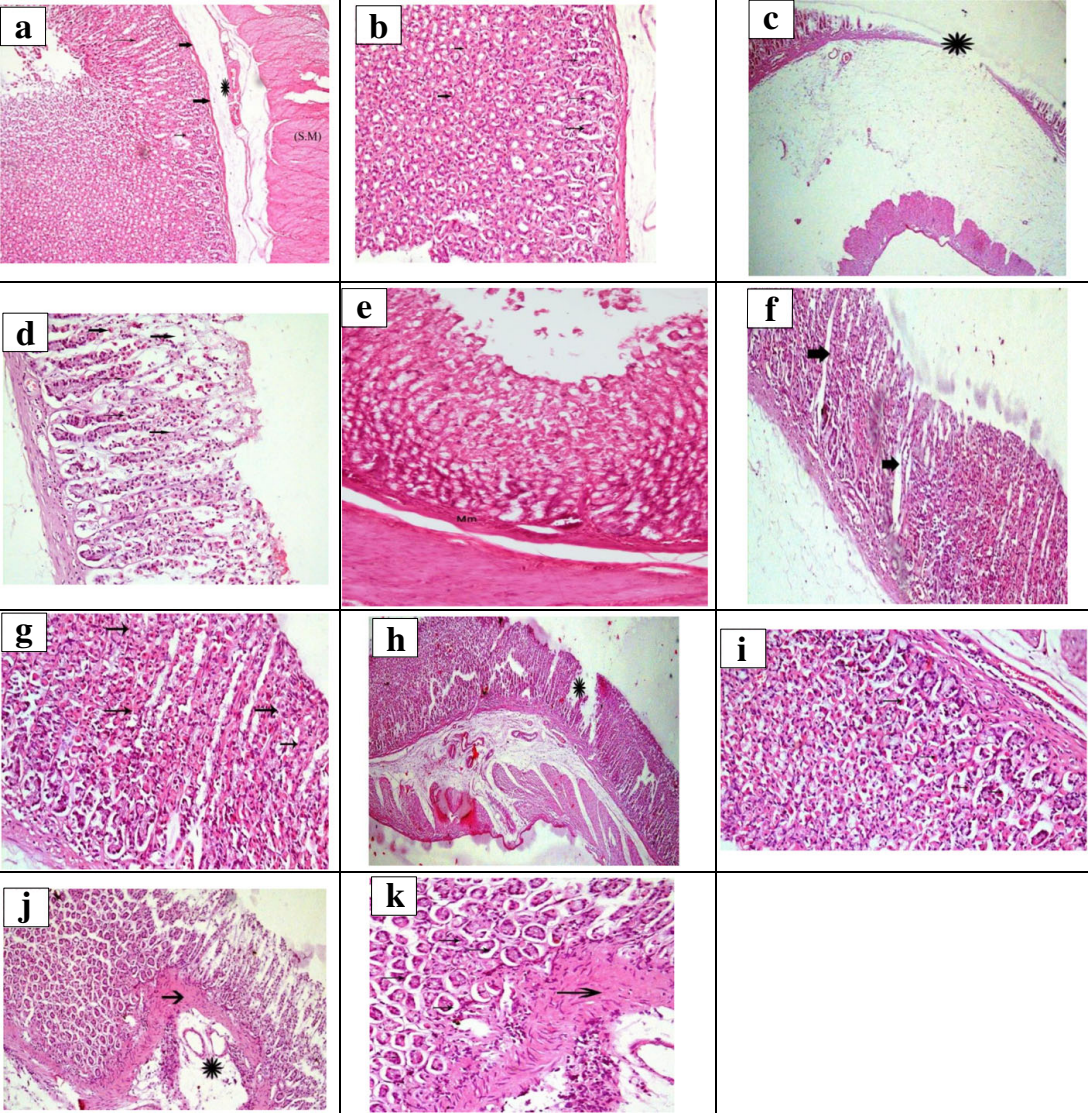
Anti gastro-ulcerogenic effect of *P. racemosa* leaves AME. **a** A photomicrograph of gastric tissue for negative control group, showed normal stomach tissue which is divided histologically into mucosal layer formed of straight tubular glands (thin arrow) then lined by the sub mucosa (thick arrow) and the connective tissue of the sub mucosa (*) finally the smooth muscle layer (S.M) (H&E X 100). **b** Higher magnification of (**a**) showed, the chief cells (thin arrow) from which mainly the glands formed with scattered parietal cells known by its eosinophilic cytoplasm (thick arrow) (H&E X 200). **c** A photomicrograph of gastric tissue for positive control (ethanol group), showed ulcerated stomach tissue with loss of most of the mucosal glands (*) (H&E X40). **d** Higher magnification of the (**c)** showed the area of ulceration with disrupted glands (thin arrow) and others are atrophied (thick arrow) (H&E X 200). **e** A photomicrograph of gastric tissue for ranitidine 50 mg/kg b.wt. treated group showed slight ulcerated and distorted mucous membrane (H&E X100). **f** A photomicrograph for of gastric tissue in rats treated with AME of *P. racemosa* leaves (125 mg/kg b.wt.) showed the ulcerated area with losing the mucosal glands (thick arrow) (H&E X 100). **g** Higher magnifications of (**f**), showed sever destruction of the mucosal glands (thin arrow) (H&E X 200). **h** A photomicrograph for of gastric tissue in rats treated with AME of *P. racemosa* leaves (250 mg/kg) showed the ulcerated area without healing features (*)(H&E X 100). **i** Higher magnification of (**h**)**,** showed exaggerated parietal cells with its eosinophilic cytoplasm (thin arrow) (H&E X 200). **j** A photomicrograph for of gastric tissue in rats treated with AME of *P. racemosa* leaves (500 mg/kg b.wt.) showed ulcerated area started to be healed (*) by formation of the granulation tissue (thick arrow) (H&E X 100). **k** Higher magnification of (**j**). showed the granulation tissue formed of connective tissue fibers and minute blood vessels formed, beneath this tissue there are gradual revolution of the glands (thin arrow) (H&E X 200)

The histopathological examination of gastric mucosa showed normal gastric mucosa in the control group which was treated with 1 mL saline (Fig. 3 a & b) while oral ethanol administration induced necrosis of apical mucosa accompanied by hemorrhage and submucosal edema (Fig. 3 c and d). Oral administration of AME in three doses induced gastro-protective effect which confirmed the results of naked eye evaluation of ulcer number and severity (Fig. 3 f and k). Administration of the AME exhibited a significant hepatoprotective effect in a dose dependent manner in paracetamol induced hepatotoxicity rats. Oral administration of a single dose of paracetamol exhibited significant elevation in serum enzyme level of ALT and AST by 46.42 and 152.6%, respectively after 24 h upon comparison with the control group. The elevated serum enzyme level in paracetamol group was significantly reduced by 56.87 & 25.16% for ALT and AST, respectively after oral administration of silymarin 25 mg/kg b.wt. AME oral administration of three dose levels (125, 250 and 500 mg/kg b.wt.) showed significant reduction in elevated serum ALT by 44.36, 55.39 and 59.3%, respectively as compared with the paracetamol treated group, while the elevated level of AST was significantly reduced at dose 125 and 250 mg/kg b.wt. by 22.74 and 27.19, respectively upon comparison with the paracetamol treated group **(**Table 7).

**Table 7:** Effects of *P. racemosa* leaves AME and silymarin on ALT and AST in paracetamol induced hepatotoxicity in rats

Item (IU/L)	Group (dose in mg/kg b.wt.)									
	Negative Control	Paracetamol640	Silymarin 25	% of change	80% AME of *P. racemosa* leaves					
					125	% of change	250	% of change	500	% of change
ALT	15.61 ± 0.65^*^	46.42 ± 1.8	20.02 ± 1.8*	56.87	25.83 ± 2.4^*@^	44.36	20.71 ± 1.58^*^	55.39	18.89 ± 1.17^*^	59.3
AST	94.77 ± 7.6^*^	152.6 ± 7.2	114.2 ± .2.0^*^	25.16	117.9 ± 2.0^*.^	22.74	111.1 ± 4.28*	27.19	138.6 ± 4.7	9.2

Values represent the mean ± S.E. Data is represented as means ± S.E; n = 8; Data were analyzed using one way analysis of variance (ANOVA) followed by Tukey Kramer multiple comparisons test. Significant at P ≤ 0.05

^*^Significant difference from positive control paracetamol group; ^@^ Significant difference from negative control group; Percent of change was calculated as regard to paracetamol control group

**Fig. 4:**
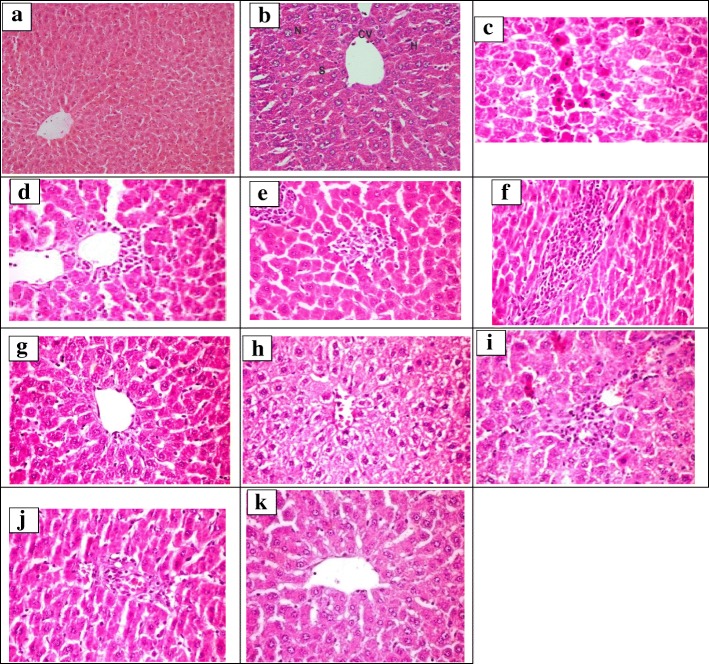
Hepatoprotective activity of *P. racemosa* leaves AME. **a, b** A photomicrograph of a control group liver section showing normal hepatic architecture with well-arranged hepatic cords, the central vein (CV) and hepatic cords of hepatocytes (H) with prominent nucleus (N) separated with blood sinusoids (S). No evidence of necrosis or inflammatory reaction in the portal area could be observed (H&E X 100 & 200). **c** A photomicrograph of positive control liver section showed disorganization of hepatic cords associated with sporadic cell necrosis (H&E X 400). **d, e** A photomicrograph of positive control liver section showed centri lobular as well as focal area of hepatocellular necrosis infiltrated by mononuclear cells (H&E X 400). **f** A photomicrograph of positive control liver section showed portal areas were intensely infiltration with inflammatory cells mostly mononuclear cells (H&E X 400). **g, h** A photomicrograph of a liver section rats treated with AME of *P. racemosa* leaves (125 mg/kg b.wt.) showed diffuse vacuolar degeneration of hepatocytes and mild portal infiltration with inflammatory cells (H&E X 400). **i** A photomicrograph of liver section treated with AME of *P. racemosa* leaves (250 mg/kg b.wt.) showed alteration nearly similar to those of the control one with focal sinusoidal dilatation and increased number of binucleated hepatocytes (H&E X 400). **j** A photomicrograph of a liver section rats treated with AME of *P. racemosa* leaves (250 mg/kg b.wt.) showed portal area appeared normal with no inflammatory cell infiltration (H&E.X.400). **k** A photomicrograph of a liver section rats treated with AME of *P. racemosa* leaves (500 mg/kg b.wt.) showed mild vacuolation of hepatocellular cytoplasm, activation of kupffer cells and increase number of binucleated hepatocytes. Portal area showed hyperplasia of biliary epithelium and formation of newly formed bile ductuoles (H&E X 400)

Histopatlogical examination for liver tissue of the control rats group showed normal architecture (Fig. 4a, b). Paracetamol administration showed granularity of the cytoplasm of hepatocytes, associated with focal hepatic necrosis, inflammatory cells infiltration and portal infiltration with leucocytes (Fig. 4c-f). A liver section of rats treated with AME of *P. racemosa* (125 mg/kg b.wt.) showed diffused vacuolar degeneration of hepatocytes and mild portal infiltration with inflammatory cells (Fig. 4 g, h). The liver section of rats treated with 250 mg/kg b.wt. revealed alteration nearly similar to those of the control. The portal area appeared normal with no inflammatory cell infiltration (Fig. 4 i, j) while liver section of rats treated with 500 mg/kg b.wt. showed mild vacuolation of hepatocellular cytoplasm, activation of kupffer cells and increase number of binucleated hepatocytes. The Portal area showed hyperplasia of biliary epithelium and formation of newly formed bile ductuoles (Fig. 4 k).

## Discussion

### Structure elucidation of the isolated compounds

The chromatographic and physical properties along with NMR spectral data of compounds 1, 2, 11 and 12 were completely in agreement with the corresponding published data for gallic acid **(1),** methyl gallate **(2),** quercetin **(11)** and ellagic acid **(12)** [27–30].

Data listed in Tables 2 and 3 for compounds 3–6 matched with the available literature for quercetin 3-*O*-α-L-arabinofuranoside (avicularin, **3**), quercetin 3-*O*-β-*D*-arbinopyranoside (**4**), quercetin 3-*O*-*β*-D-glucopyranoside (**5**) and quercetin 3-*O*-α-L-rhamnopyranoside (quercetrin, **6**) (27, 29–32].

**Compound 7:** It gave the chromatographic properties and UV data characteristic for luteolin-7-*O*- glycoside as well as it gave D**-**glucose and luteolin upon complete acid hydrolysis. Its ^1^H-NMR showed an ABX spin coupling system of three aromatic protons, H- 2’/6’ (*m*, δ 7.45) and 5’(*d*, δ 6.90) together with singlet signal at δ 6.79 for H-3 characteristic for the luteolin nucleus. Down field shift of H-6 (δ 6.45) and H-8 (δ 6.77) together with doublet of H- 1″ at 5.09 revealed the presence of sugar moiety at 7-OH. Final confirmation of **7** was done through its ^13^C-NMR data which revealed the presence of 15 carbons resonance for luteolin nucleus especially for C-3 (103.64) as well as the characteristic position of C-7 resonance (162.86 ppm) together with the six carbons for glucose moiety which confirmed the location of glucose moiety at C-7 [31, 32].

**Compound 8:** It showed the chromatographic characters and UV data for ellagitannins [33]. On complete acid hydrolysis, ellagic and gallic acids were liberated into the organic layer while glucose was detected in the aqueous layer (CO-PC). ^1^H-NMR of **8** showed two singlets each for one proton at δ 6.42 and 6.40 for H-3′′′ and H-3′′ of hexahydroxydiphenoyl (HHDP) group, together with singlet signal for two protons H-2′/6′ of galloyl moiety in the range at 6.97 ppm. The sugar moiety was confirmed as β-^4^C_1_ glucose from the doublet signal for H-1at δ 5.70 (8.7 Hz) [33–35]. Downfield shift of H-1, H-6_a_ and H-4 at 5.70, 4.86 and 4.60, respectively gave an evidence for attachment of galloyl moiety on OH-1 and bi-functioned esterification of both OH-4 and OH-6 with the HHDP moiety because of the anisotropic effect of the deshielding cone of the upper phenyl ring of HHDP on H-6a. Further confirmation of the structure was achieved via ^13^C-NMR data which revealed the downfield of C-1 (94.73) and up field of C-2 (71.61) due to the galloylation of OH-1. Moreover, according to ^13^C-substituent additive rules, esterification of OH-6 and OH-4 with HHDP was confirmed from the downfield of C-4 (71.61) and C-6 (62.74) as well as upfield shift of C-3 (73.82) and C-5 (73.33) [36]. The presence of galloyl group was established from the presence of five carbon atoms characteristic for gallic acid. Moreover, the resonance of 14 carbon atoms revealed the presence of HHDP moiety. Therefore, compound **8** was identified as 1-*O*-galloyl-4, 6-(*S*)-hexahydroxydiphenoyl-*β*-D-glucopyranose (Strictinin).

**Compound 9:** It exhibited the chromatographic properties and the very broad strong UV absorption maxima (290 nm) characteristic for C-glycosidic ellagitannin. Moreover, it gave ellagic acid in the organic phase with unknown intermediate ellagitannin on complete acid hydrolysis in addition to the absence of glucose in the aqueous phase which gave the conformation of the C-glycosidic nature of **9** [35]. ^1^H-NMR data revealed the presence of two singlets signals one of which for two proton at δ 6.69 ppm and the other for one proton at δ 6.50 ppm characteristic for a C-glycosidic flavogallonoyl group attached to C-1, C-2, C-3 and C-5 and HHDP at C-4 and C-6 of an open chain glucose. C-glycosidic structure of **9** was established from the δ ppm and J-values of the glucose moiety, especially that of H-1 at δ 5.51 (4.0 Hz), which confirmed the full substituted open glucose with anomeric axial OH-group [35, 37, 38]. More conformation of the compound was done from its ^13^C-NMR data which showed 14 carbon resonances for HHDP moiety together with 21 carbon resonance characteristic for flavogallonoyl units attached by C-glycosidic linkage with C-1 of glucose. The open chain glucose unit instead of hemiacetal ^4^C_1_–pyranose was clearly proved due to the strong upfield location of C-1 at δ 66.35 in comparison to that of pyranose form (+ ≈ 90–95 ppm). Moreover, the downfield shift of C-3’ at δ 113.78 (≈ + 10 ppm) and upfield shift of the carbonyl carbon C-7’ at 164.96 (≈ − 6 ppm) of the flavogallonoyl moiety gave more conformation about the C-glycosidic nature. The δ ppm and J_12_- values of H-1 glucose proved that the absolute configuration at the anomeric position should be of axial OH and equatorial H. Additionally, UPLC-ESI-MS negative mode spectrum of **9** showed pseudo-molecular ion peaks at *m/z* 933 assigned to [M-H]^−^ which matched with the reported molecular weight (934) of castalagin [39]. Accordingly, compound **9** was identified as 2,3,5-(*S*)-flavogalonoyl-4,6-(*S*)-hexahydroxydiphenoyl-D-glucose (Castalagin) [35, 37, 38].

**Compound 10:** It showed the chromatographic properties and UV absorption characteristic for C-glycosidic ellagitannin. *C*-glycosidic structure of **10** was expected from its complete acid hydrolysis which gave ellagic acid with unknown intermediate ellagitannins as well as the absence of glucose in the aqueous hydrolysate (Co-PC), [35]. Its ^1^H-NMR explained its identity as an equimolecular mixture [40]. In the aromatic region, eight singlets integrated in total to three protons, two of which for HHDP group (H-6″″/6′″″) at δ ppm 6.63, 6.61, 6.62, 6.59 and the 3rd one for H-6′″ at 7.32, 6.79, 6.79, 6.78 (1H in total, each s) assigned to one C-glycosidic flavogallonoyl moiety in four stereoisomers. Additionally, the repetition of some sugar resonances in the aliphatic region as H-1 at δ ppm 4.01 and 3.98 appeared as two br s signals which supported the presence of the equimolecular isomeric mixture. Appearance of H-1 as br s signals together with J-values of the other resolved resonances was approved with the stereo structure of this moiety in previously reported grandinin [41]. The four stereoisomers mixture for **10** was established from the presence of carbon resonance at δ ppm 101.15, 101.07 and 99.42, 99.34 which attributed to the hemiketal carbon resonance of the pentose moiety with carbon resonance at 47.03, 46.96 and 45.86 for C-1 due to isomerism of the pentose moiety between pyranose and furanose as well as anomeric equilibria in both conformers of sugars. The presence of pentopyranose and pentofuranose equilibrium was evidenced also from the C-4 pentose resonance as four signals at δ ppm 80.06, 79.3, 72.09 and 69.10 indicative for the four stereoisomers mixture. All the remaining resonances of ^1^H and ^13^C NMR spectra of **10** were assigned by comparison with previously published data on grandinin [40, 41]. Finally, UPLC-ESI-MS negative mode spectrum of **10** showed pseudo-molecular ion peaks at *m/z* 1065 assigned to [M-H]^−^ which confirmed the molecular weight of grandinin (1066) as reported before [38].

### Biological activities

Based on chemical and spectroscopic evidences, the present study showed that AME of *P. racemosa* leaves contains flavonol aglycones, flavonol glycosides, flavone glycosides, phenolic acids and hydrolysable tannins, which gave gallic and ellagic acids degradation products upon hydrolysis.

AME showed significant antioxidant activities as indicated by its high DPPH scavenging percentage (89.67%) and low SC_50_ value (4.6 μg/mL), which is mainly attributed to its constituents. Phenolic and flavonoids are responsible for a wide variety of pharmacological activities [42], which depend on their structure as antiulcer [43], analgesic anti-inflammatory [44], antioxidant [45] and hepatoprotective [46]. Their biological activities were dependent on the degree of hydroxylation, substitutions and conjugations as well as the degree of polymerization [47]. Most of the potential health benefits of flavonoids arise from their antioxidant activities that are mediated by hydroxyl groups in flavonoids which scavenge free radicals and/or by chelate metal ions [48].

Hydrolysable tannins are considered as outstanding antioxidant agents due to the presence of several gallic and hexahydroxy diphenyl groups which possess the abilities to provide protons and formation of stable free radicals which enables them to be the major active groups in the tannins molecule [49, 50]. Moreover, the linkage between the monomers of hydrolysable tannins and phenolic hydroxyl groups as well as the esteric and the glycosidic bond also plays an important role in their antioxidants properties. The antioxidant properties of ellagitannins are responsible for their different biological activities as antiulcer [51], hepatoprotective [52] as well as anti-inflammatory [50]. It is worthy to notice that the activity can be attributed not only to phenolic compounds but also to other constituents that may participate by a role or exhibit synergistic effect as reported for other *Pimenta* species where the methanol extract of *P. racemosa* var. *ozua* leaves showed significant anti-inflammatory activity against acute edema, both when taken orally and topically [53] while its aqueous extract exhibited antinociceptive and anti-inflammatory activities [7]. Additionally, the anti-inflammatory activity of abietic acid isolated from *P. racemosa* var. *grissea* was proved [54].

Similarly, anti-inflammatory, analgesic, antipyretic and gastric antiulcer activities were proven for aqueous suspension of *P. dioica* (L.) in animal models where combination of anti-inflammatory and antiulcer activity represented a remarkable discovery for the ideal anti-inflammatory agent without the potential adverse effects on the gastrointestinal tract [55] which matched with our results for AME of *P. racemosa* leaves. Additionally, the antiulcerogenic and hepatoprotective activities were proved for other member of Myrtaceae by using animal models [56].

## Conclusion

This paper proved the isolation and identification of phenolic constituents of AME of *P. racemosa* leaves in addition to its activities as antioxidant, analgesic anti-inflammatory, antinociceptive, gastro and hepatoprotective. This is hopeful for further phytochemical and biological investigations to confirm the possibility of their therapeutic effects, which may be important for the development of new natural drugs.

## Abbreviations

ALT: Alanine aminotransferase
AME: Aqueous methanol extract
AST: Aspartate aminotransferase
DPPH: 2,2-Diphenyl-1-picrylhydrazyl
PI: Percentage inhibition
SC_50_: The concentration required to scavenge DPPH by 50%
UPLC-ESI-MS: Ultra performance liquid chromatography-Electrospray ionization-Mass spectrometry
